# Effectiveness of ultrasound (US) and slightly acidic electrolyzed water (SAEW) treatments for removing *Listeria monocytogenes* biofilms

**DOI:** 10.1016/j.ultsonch.2024.107190

**Published:** 2024-12-10

**Authors:** Hongrui Ren, Yu Quan, Shaokang Liu, Jianxiong Hao

**Affiliations:** College of Food Science and Biology, Hebei University of Science and Technology, No. 26 Yuxiang Street, Shijiazhuang 050018, China

**Keywords:** *Listeria monocytogenes*, Biofilm, Slightly acidic electrolyzed water, Ultrasound

## Abstract

Biofilms can persist in food industry environments leading to repeated cross-contamination, thus threatening human health. Slightly acid electrolyzed water (SAEW) and ultrasound have emerged as environmentally-friendly antimicrobial agents. However, their bactericidal efficacy is not high when used alone. In the present study, the effect of ultrasound combined with SAEW on removing *Listeria monocytogenes* biofilms from a glass surface was evaluated. *Listeria monocytogenes* biofilms were treated by immersion in sterilized deionized water (control treatment), immersion in sterilized deionized water combined with ultrasound (US treatment), immersion in SAEW (SAEW treatment), immersion in SAEW combined with ultrasound treatment (SAEW + US treatment), immersion in SAEW followed by immersion in sterilized deionized water combined with ultrasound (SAEW − US treatment), and immersion in sterilized deionized water combined with ultrasound followed by immersion in SAEW (US − SAEW treatment). The results showed that treating biofilms by US − SAEW treatment led to the lowest number of *Listeria monocytogenes*, the lowest amount of biofilm remaining on the glass slide were visualized by crystal violet staining and scanning electron microscope, and a minimal content of extracellular polymeric substances. Overall, the highest biofilm removal efficacy was observed for the US − SAEW treatment.

## Highlights

• US − SAEW treatment can effectively cleaned up all *Listeria monocytogenes* biofilms at 1 min.
• US − SAEW effectively decreased extracellular polymeric substance.
• US − SAEW had higher biofilm clearance efficacy than SAEW and US alone.

## 1 Introduction

*Listeria monocytogenes* (Lm), a Gram-positive rod-shaped facultative anaerobe, is an important food-borne pathogen capable of causing listeriosis [1]. Listeriosis is a rare food-borne disease with both invasive and non-invasive forms that is associated with high mortality [2]. Over the years, listeriosis has been one of the main food-borne diseases causing public health problems for humans and animals worldwide [3]. The changes in globalized food production systems and the growing use of minimally-processed have increased the incidence of listeriosis in recent years [4]. Infection with Lm can cause symptoms such as fever, dyspnea, spontaneous bacterial peritonitis [5], brain abscesses [6] and meningitis [7]. It particularly affects the elderly and immunocompromised individuals, who are highly vulnerable to infection, and in whom severe cases can be fatal [8]. In pregnant women, infection with Lm may lead to stillbirth, miscarriage, or fetal death [9]. Lm can adhere to biological or non-biological surfaces, secrete sticky extracellular polymeric substances (EPS) and proliferate, forming multi-layered biofilms with specific structures [10]. The process of biofilm formation involves several sequential phases, including bacterial adhesion, the production of EPS, bacterial micro-colonization, the formation of mature biofilm, and dispersion [11]. The EPS of mature biofilm primarily contain extracellular polysaccharides, extracellular proteins, and extracellular DNA, and are considered to be key factors in protecting cells from adverse environmental conditions [12]. Bacterial cells in biofilms are 10–1000 times more resistant to disinfectants and antimicrobials than their planktonic counterparts [13]. Mature biofilms can provide stable ecological niches for Lm cells by protecting them from disinfectants and other stressors. This increases the chances of Lm surviving in the processing plants [14], [15], [16]. Therefore, it is important to select an efficient and environmentally friendly biofilm removal method.

Currently, methods for removing Lm biofilms include heat treatment [17], enzymes [18], [19], and ozone [20]. However, the duration and temperature of heat treatments must be controlled or else the quality of the product will be affected. The current high cost of enzymes greatly limits their practical use [21]. High concentrations of ozone can be toxic and harmful to human health, and its long-term use may also have corrosive effects on certain materials [20], [22]. Therefore, the use of these methods in the food industry is still limited. Electrolyzed water is a general term for acidic and alkaline water obtained through the electrolysis of diluted hydrochloric acid or saltwater using an electrolyzed water generator. In recent years, slightly acidic electrolyzed water (SAEW) has been used in the field of food processing due to the advantages of instant effect against a broad spectrum of microorganisms, high efficiency and safety, no residue or other sequelae, and no irritation of human skin. It has been reported that SAEW effectively kills bacteria and spores such as those from *Staphylococcus* and *Bacillus subtilis* [23]. However, the use of SAEW alone for biofilm removal is limited by the presence of EPS. Ultrasound (US) is an emerging physical sterilization technology. US refers to the sound wave with a frequency greater than 20 kHz, which produces a cavitation effect on microbial cells and then plays a bactericidal role [24]. Cavitation generates mechanical effects which can disrupt the membranes of bacterial cells and allow some substances to pass from the medium into the cells [25]. Cavitation may also damage the structure of cellular organelles, promoting the release of cathepsin enzymes from the lysosome and calcium from the sarcoplasmic reticulum. This in turn activates the calpain enzyme system and accelerates proteolysis [26]. As a non-thermal sanitization technology, US has the advantages of being a non-energy intensive technique requiring only a short treatment duration, and it has minimal effects on food flavor and nutrient loss [27]. It also has a wide potential for application in the field of food sanitization. US can also be used as an adjunctive technique to chemical treatment, as it enhances the penetration of chemical inhibitors into the biofilm and further improves its eradication. It was reported that the use of US in combination with SAEW during precooling was effectively in reducing the bacteria population in chicken breasts [28].

However, there are no reports of US being used in combination with SAEW to remove Lm biofilms. In the present study, we compared the efficacy of treatments for inactivating Lm cells and biofilms using: (1) sterilized deionized water immersion, (2) sterilized deionized water immersion combined with US, (3) SAEW immersion, (4) SAEW immersion combined with US treatment, (5) SAEW immersion followed by sterilized deionized water immersion combined with US, and (6) sterilized deionized water immersion combined with US followed by SAEW immersion. Crystal violet staining and scanning electron microscopy (SEM) was used to visualize the appearance and ultrastructure of the bacteria and to reveal the combined effects of US and SAEW on the morphology of Lm biofilms. Additionally, the changes in EPS content were observed. In the present study, an environmentally friendly, efficient and cost-effective sterilization technology is discussed. And it would provide a method to sterilize future utensils in the food industry, such as glass packaging materials or glass drinking utensils.

## 2 Materials and methods

### 2.1 Preparation of Lm biofilms

The strain used in this study was Lm ATCC 19114, which was purchased from Beijing Solarbio Science & Technology Co., Ltd (Beijing, China). The strain plates were marked and then cultured at 37 °C for 24 h. A single colony was then selected, placed in 100 mL of sterile liquid medium, and cultured at 37 °C for 24 h with rotation at 160 rpm. The number of viable bacteria was about 10^8^–10^9^ CFU/mL. First, the glass slides to be used were cleaned by soaking them in acetone for 15 min to remove grease and then rinsing them with deionized water. Then they were soaked in ultra-pure water for 15 min, dried, and sterilized at high temperature for later use. The sterile slides were then placed in culture dishes and 1 mL of the bacterial solution was added to each slide along with 25 mL of trypticase soy-yeast extract broth liquid medium. The bacteria were cultured in a constant-temperature incubator for 5 d, during which time the liquid was changed on alternate days until the biofilm had matured.

### 2.2 Ultrasound treatment

For the US treatment, the samples were placed in centrifuge tubes (50 mL) containing 45 mL of sterilized deionized water. The tubes were then placed in an ultrasonic bath (XM-P222H, Kunshan Ultrasonic Instrument Co., Ltd., Kunshan, China) for 15, 30, 45, or 60 s. For the SAEW + US treatment, the sterilized deionized water was replaced with previously prepared SAEW and the other methods used were the same as those described above. The frequency and power of the ultrasound bath were 24 kHz and 420 W, respectively. During the whole process, the temperature of the ultrasonic bath was kept constant at approximately 25 °C by adding fresh cold water as necessary.

### 2.3 Preparation of electrolyzed water

A diaphragm-free electrolysis cell developed in our laboratory was used to prepare SAEW. Briefly, 4 L of tap water was placed in the cell, and then 12.0 g of NaCl and 0.5 mL of concentrated HCl were added and thoroughly mixed. After connecting the power supply, the SAEW was produced in the electrolysis cell under a stable current of 1 A.

A pH meter (Suntex SP-701, Suntex Instruments Co., Ltd. Taipei, Taiwan) was used to measure the pH value. The concentration of free chlorine was measured using iodometry.

### 2.4 Treatment of Lm biofilms

The Lm biofilms were cultured for 5 d and then any floating bacteria were removed by rinsing with sterile phosphate buffered saline. The remaining biofilms were treated using different techniques to test their effectiveness in eliminating the bacteria. The CK sample was the Lm biofilms which were cultures for 5 d. The control group samples were only immersed in sterilized deionized water. The US samples were immersed in sterilized deionized water and exposed to US at 24 kHz. The SAEW samples were only immersed in SAEW. The SAEW + US samples were immersed in SAEW and exposed to US at 24 kHz. The SAEW − US samples were first immersed in SAEW and then in sterilized deionized water and exposed to US at 24 kHz. The US − SAEW samples were first immersed in sterilized deionized water and exposed to US at 24 kHz before being immersed in SAEW. To determine the biomass of the Lm biofilms, the treated biofilm carriers were removed then rinsed several times with sterile phosphate buffered saline solution. After washing away the impurities and any planktonic bacteria present, the biofilm on the front of each carrier was scraped off using a cell scraper and then transferred to a test tube containing 9 mL of normal saline. After gradient dilution, 0.1 mL was coated on the plate, which was then placed in a constant-temperature incubator and cultured at 37 °C for 24 h. Then the colonies were counted, and their numbers were expressed as log CFU/mL. Three parallel experiments were performed for each treatment, and the average value was recorded.

### 2.5 Observation of Lm biofilms by crystal violet staining

Each biofilm carrier was removed, cleaned with phosphate buffered saline, and fixed with 20 mL of methanol for 15 min before being stained with 20 mL of 2 % crystal violet for 5 min. After rinsing with ultra-pure water, the biofilm carrier was dried at 30 °C for 30 min. Then, 20 mL of 33 % glacial acetic acid were added for decolorization. After drying, the sample was placed under an optical microscope for observation. Three evenly distributed fields of view were randomly selected for each sample, and each treatment was repeated in triplicate.

### 2.6 Observation of Lm biofilms by scanning electron microscope

Each biofilm carrier was cultured for 5 d, cleaned with phosphate buffered saline, fixed overnight with 2.5 % glutaraldehyde in phosphate buffered saline, and then rinsed with phosphate buffered saline. After dehydration steps with 50 %, 70 %, 80 % and 90 % ethanol for 10 min, the samples were dehydrated twice with anhydrous ethanol for 15 min, and then dehydrated twice with isoamyl acetate for 15 min. After freeze-drying for 24 h, the samples were sprayed with gold and then observed using a scanning electron microscope. Three evenly distributed fields of view were randomly selected for each sample, and each treatment was repeated in triplicate.

### 2.7 Extraction of bacterial EPS

Biofilm-carrying slides were treated with fungicide and placed in Petri dishes, and then 5 mL of deionized water was added to each dish. The biofilm on both sides of the slides was scraped off using a cell scraper and collected in 10-mL centrifuge tubes. The tubes were oscillated at 150 rpm at 25 °C for 1 h before being placed in a water bath at 60 °C for 1 h. Then, they were centrifuged at 12,000 × *g* for 20 min at 4 °C. The resulting supernatants contained the extracted bacterial EPS [29].

### 2.8 Determination of extracellular proteins in the biofilms

The Lm biofilms were cultured on glass slides in Petri dishes at 37 °C for 5 d with the liquid being changed every other day. After the Lm biofilms were exposed to the experimental treatments, the components of the EPS were identified. The Lm biofilms from each treatment group were extracted as described in 2.7. The absorbance values were measured at 562 nm in microplate reader (SpectraMax M2, Molecular Devices Co., Shanghai, China) according to the steps of the total protein assay kit (Nanjing Jiancheng Bioengineering Institute, Nanjing, China). Then the concentration of the sample was calculated by BCA protein concentration assay. Three parallel experiments were performed for each treatment, and the average value was recorded.

### 2.9 Determination of extracellular polysaccharides in biofilms

The Lm biofilms were cultured on vitreous carrier plates at 37 °C in culture dishes for 5 d, and the liquid was changed every other day. After Lm biofilms were exposed to the experimental treatments, the EPS samples from the Lm biofilms from each treatment group were extracted as described in 2.7. The remaining polysaccharides in the EPS were determined using phenol–sulfuric acid colorimetry, and the polysaccharide content was calculated using a standard curve. Three parallel experiments were performed for each treatment, and the average value was recorded.

### 2.10 Determination of extracellular DNA in the biofilms

The Lm biofilms were cultured at 37 °C on glass slides in culture dishes for 5 d, and the liquid was changed on alternate days. After the Lm biofilms were exposed to the experimental treatments, the EPS of the Lm biofilm from each treatment group were extracted as described in 2.7, and the absorbance value was determined at 260 nm using a microplate reader (SpectraMax M2, Molecular Devices Co., Shanghai, China). Three parallel experiments were performed for each treatment, and the average value was recorded.

### 2.11 Statistical analysis

Each treatment was repeated three times. For each treatment, data from independent replicate trials were pooled and the means and standard deviations were calculated. All data were analyzed using Duncan’s multiple range test (SPSS16.0 for Windows, SPSS Inc., Chicago, IL, USA). Significant differences between treatments were established at a significance level of *p* < 0.05. Charts were drawn using Origin software (OriginPro 2018, OriginLab, Northampton, MA, USA).

## 3 Results and discussion

### 3.1 Comparing effect of each treatment on cleaning biofilms

After the Lm biofilm samples were treated using the six experimental groups for different lengths of time, the bactericidal effect of each treatment method and its effect on the Lm biofilms was evaluated using the plate coating method [30].

**Fig. 1:**
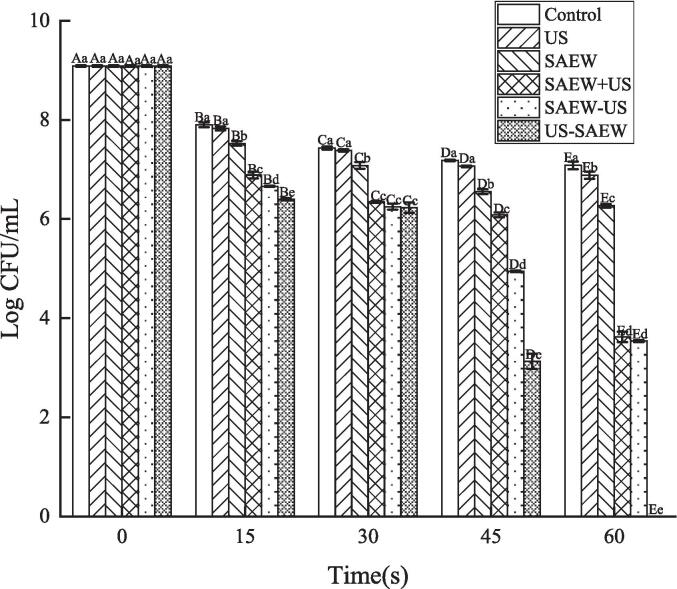
The bactericidal effect of different treatments on *Listeria monocytogenes* biofilms. Capital letters represent significant differences between different processing times. Lower case letters represent significant differences between different treatments (P < 0.05). US, ultrasound. SAEW, slightly acidic electrolyzed water.

Compared with the initial count of 9.09 log CFU/mL, the number of Lm CFUs decreased as the treatment time increased. After the 15 s treatments, the control group bactericidal rate was 13.09 %. After US treatment for the same length of time, the bactericidal rate was 13.86 %, which was not significantly different from the control treatment (P > 0.05). The Lm bactericidal rate was 17.20 % after the SAEW treatment. The SAEW + US treatment Lm bactericidal rate was 24.20 %. The SAEW − US treatment Lm bactericidal rate was 26.73 %. The bactericidal rate was 29.56 % after the US − SAEW treatment. There were significant differences between these four treatment methods. At 30 s, the control and US groups bactericidal rate were 18.15 % and 18.81 %, respectively. There were no significant differences between the two treatments. The SAEW treatment Lm bactericidal rate was 22.11 %, which was significantly different from the control and US groups (P < 0.05). The SAEW + US, SAEW − US and US − SAEW treatments had bactericidal rates of 29.15 %, 31.24 % and 31.46 %, respectively, but the differences were not significant (P > 0.05). At 45 s, control group bactericidal rate was 20.98 %, compared with 22.26 % after US treatment, which was an insignificant difference (P > 0.05). The SAEW treatment bactericidal rate reached 27.91 %. The SAEW + US treatment bactericidal rate was 33.00 %. The SAEW − US treatment bactericidal rate was 45.58 %. The bactericidal rate reached 65.60 % after US − SAEW treatment. There were significant differences between these four treatment methods. At 60 s, there were significant differences between the five methods (P < 0.05), and the bactericidal rate for the US-SAEW was 100.0 % (Fig. 1).

Jia et al. found that, after US − SAEW treatment, the total bacteria of crayfish decreased by 1.48 lg CFU/g compared with the blank group, indicating a stronger bactericidal effect compared with the single treatment [31]. Weiqing et al. found that, US − SAEW could inhibit the total number of colonies and the growth of psychrophile bacteria in sea bass meat samples during storage, and extend the shelf life for 6 days [32]. These are similar to the results of our study. Possibly because the structure of the Lm biofilm and the cell walls of the Lm cells had been destroyed by the ultrasound treatment. Thus, the SAEW could easily penetrate the cell wall and kill the microorganisms more rapidly. Although the total number of colonies results proved that the removal rate of US − SAEW was the highest among the six groups, the biofilm removal effect still needed to be further verified. Thus, in the next section, the reduction of Lm biofilm during each treatment will be observed by microscopy.

### 3.2 Observation of Lm using crystal violet staining

**Fig. 2:**
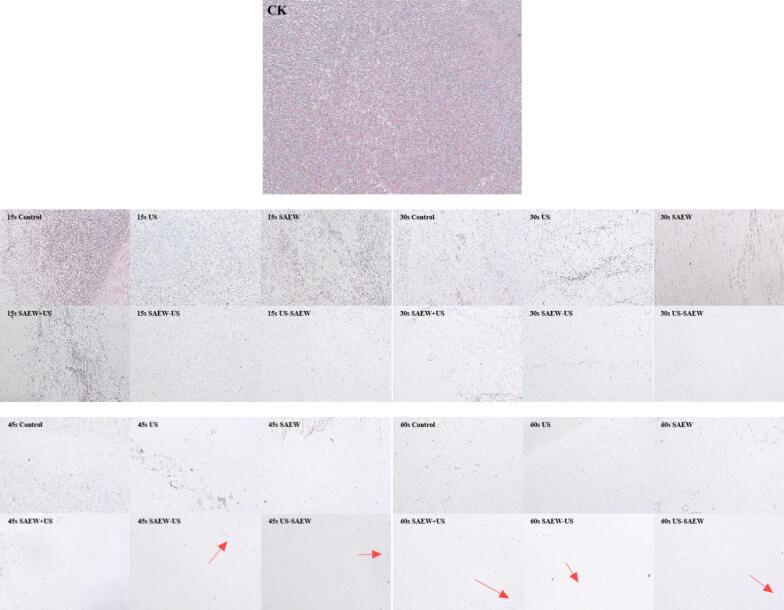
Observation of *Listeria monocytogenes* biofilm by crystal violet staining. US, ultrasound. SAEW, slightly acidic electrolyzed water. CK represents *Listeria monocytogenes* biofilm cultured for 5d.

The effect of different treatments on reducing the amount of Lm biofilm was observed using crystal violet staining. Crystal violet, as an alkaline dye, can bind to the extracellular matrix components of the biofilm and the bacteria themselves (whether living or dead bacteria) and stain them purple. In Fig. 2, CK is the local observation photograph of a Lm biofilm cultured for 5 d, and it has many local bacterial sheets. With regard to the 15 s treatments, the area of the purple region decreased after the control, US, SAEW, and SAEW + US treatments. However, many purple areas were still clearly visible, indicating the persistence of many dense biofilms. This showed that the clearing effect of these treatments was poor. After the SAEW − US and US − SAEW treatments, no obvious lamellar structures were observed in the purple region, indicating that the number of biofilms had been significantly reduced and that the biofilms had been cleared by these two methods. However, there were significantly fewer purple areas after the US − SAEW treatment. As for the 30 s treatments, some dense purple areas were visible after the control and US treatments. A few dense purple areas were visible after the SAEW and SAEW + US treatments. After the SAEW − US and US − SAEW treatments, no obvious dense purple areas were seen. Again, the US − SAEW treatment exhibited significantly fewer purple areas. This indicated that treatment with US followed by immersion in SAEW was the most effective of the six treatments for removing biofilm. After the 45 s treatments, the control group still exhibited a large area of purple residue. US, SAEW, SAEW + US, SAEW − US and US − SAEW treatments still showed small areas of purple residue, where US − SAEW treatment resulted in the least amount of residue, indicating the best removal effect. After the 60 s treatments, there was a significant reduction in the number of biofilms present for all six treatments, especially in the US − SAEW treatment, where there was almost no purple residue. Crystal violet staining showed a significant reduction in purple area after US − SAEW treatment, indicating that the biofilm was removed, and the results were consistent with those of the total number of colonies. After crystal violet staining, it was preliminarily observed that the clearance effect of the biofilm treated with US − SAEW was the best among these treatment groups. In order to further observe the changes in the status of bacteria during the removal of the biofilm more clearly, scanning electron microscopy was performed.

### 3.3 Observation of Lm biofilms using a scanning electron microscope

**Fig. 3:**
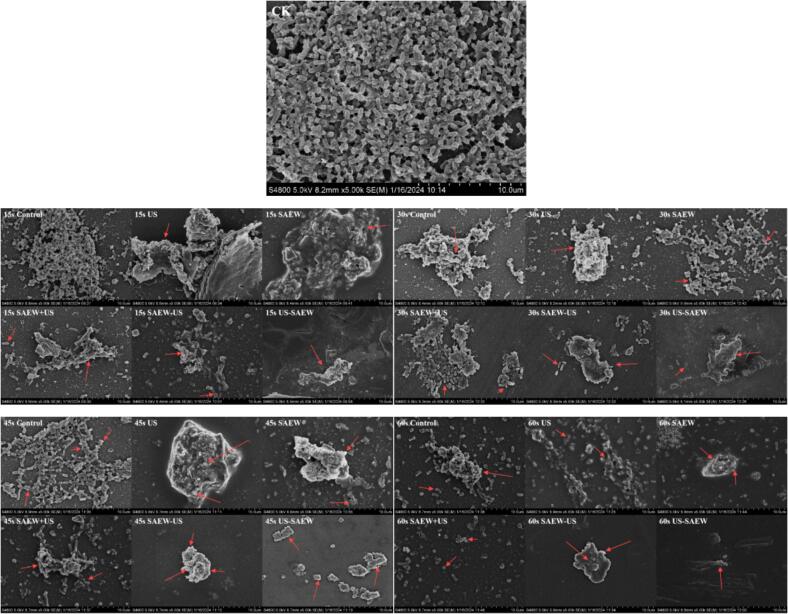
Observation of *Listeria monocytogenes* biofilm by crystal violet staining. US, ultrasound. SAEW, slightly acidic electrolyzed water. CK represents *Listeria monocytogenes* biofilm cultured for 5d.

The morphological changes in the Lm biofilms after the treatments were also observed using scanning electron microscopy. In Fig. 3, CK was a biofilm cultured for 5 d. It was observed that many bacteria had adhered to the surface of the carrier, but the cells were complete, full, and not destroyed. In addition, there was a large amount of extracellular matrix between the cells. After the control treatment, the amount of biofilm decreased as the treatment time increased. Individual cells were broken and a smaller amount of extracellular matrix was present between the cells, but no significant deformation occurred. As a result of the treatments, the amount of biofilm and intercellular extracellular matrix was significantly reduced, and the cells were ruptured and deformed in all experimental groups. The number of both bacteria and extracellular matrix decreased with increasing treatment time and there was only a small amount of extracellular matrix with no visible cellular residue after 60 s of US − SAEW treatment. It was found that, there was less EPS on the outside of the isolated individual bacteria and a thinner EPS layer compared to the biofilm, which would allow greater penetration of SAEW. This is in agreement with the results of Cai et al. that *Pseudomonas fluorescens* biofilm treated with acidic electrolyzed water showed significant indentations and cell aberrations appear on the cell surface [33]. This indicates that US − SAEW treatment has a high removal rate of biofilm, which may be due to the fact that ultrasonic cavitation destroys the cell wall of bacteria in the microbial coating, increases the permeability of cell cells, and speeds up the leakage of cell contents. In this way, the interaction area between SAEW and bacteria is increased, making it easier for SAEW to penetrate the cell wall, and the adhesion ability of biofilm is weakened, causing the biofilm to remove or the bacteria to die. In order to preliminarily explore the mechanism of biofilm removal, we measured the changes of extracellular polymers during biofilm being treated.

### 3.4 Changes in extracellular polymers during biofilm removal

**Fig. 4:**
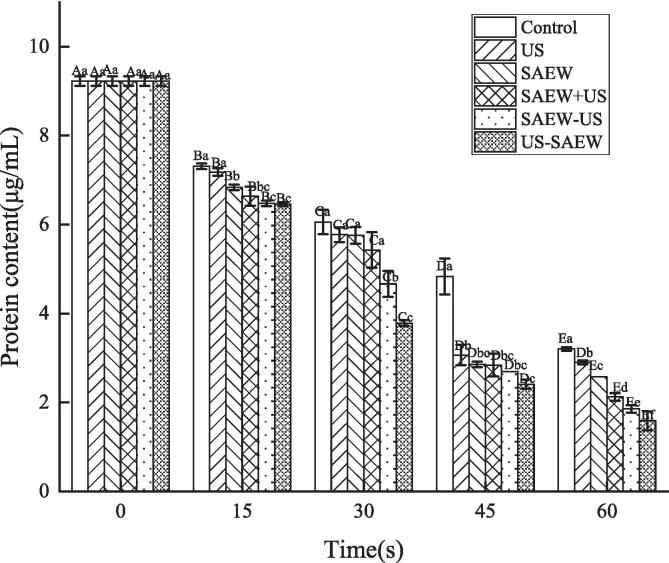
The changes in extracellular protein of *Listeria monocytogenes* biofilms after different treatments. Capital letters represent significant differences between different processing times. Lower case letters represent significant differences between different treatments (P < 0.05). US, ultrasound. SAEW, slightly acidic electrolyzed water.

The EPS of biofilm is composed of proteins, polysaccharides, nucleic acids and lipids, among which extracellular DNA (eDNA) and biofilm-related proteins play an important role in intercellular communication [34], [35], [36]. Extracellular proteins are the most abundant part of extracellular polymers in biofilms. With increasing treatment time, the amount of extracellular protein decreased, and the adhesion between bacteria was reduced. After the control, SAEW, SAEW + US, SAEW − US and US − SAEW treatments, the extracellular protein content gradually decreased from an initial content of 9.23 μg/mL with a significant decrease as the treatment time increased (P < 0.05). Although there was no significant difference between the 45 s and 60 s treatments in the US group, the extracellular protein content decreased with increasing treatment time. For the different treatment times, the extracellular protein content in the US − SAEW treatment group was the lowest after 60 s of treatment, at 1.53 μg/mL (Fig. 4). The EPS of biofilms contain many multifunctional proteins, which play an important role in biofilm adhesion [37]. This showed that the US − SAEW treatment group was most effective at destroying extracellular proteins, and the gradual decrease in protein content indicated that the biofilm adhesion was indeed reduced. This resulted in the disruption and removal of biofilms and a trend towards a reduction in extracellular proteins which is consistent with the previous results.

**Fig. 5:**
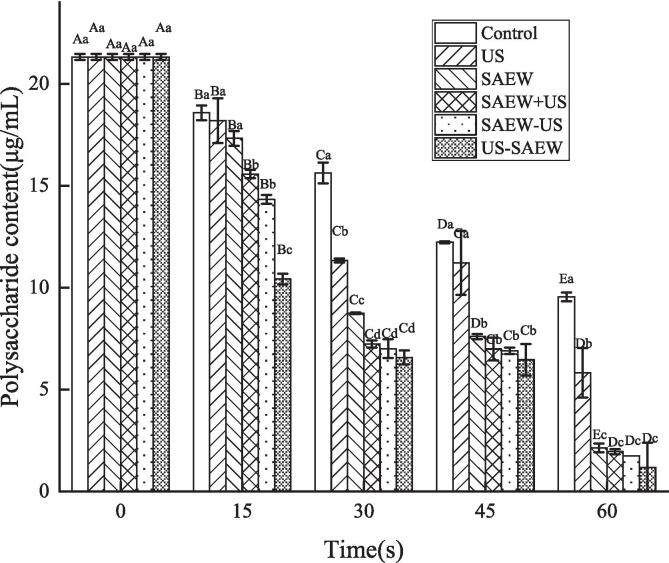
The changes in extracellular polysaccharide of *Listeria monocytogenes* biofilms after different treatments. Capital letters represent significant differences between different processing times. Lower case letters represent significant differences between different treatments (P < 0.05). US, ultrasound. SAEW, slightly acidic electolyzed water.

Extracellular polysaccharide is an important component of the extracellular polymer of bacterial biofilm, which can increase the thickness and hardness of the biofilm, promote the adhesion of bacteria to biological and other abiotic surfaces, establish and maintain the biofilm structure. Extracellular polysaccharide is the main reason for bacterial adhesion to contact surfaces. It can also increase the resistance of biofilms and enhance the ability of bacteria to infect host cells [37]. Therefore, it is particularly important to degrade exopolysaccharides when removing biofilms. Fig. 5 shows that with increasing treatment time, the content of extracellular polysaccharide decreased from an initial level of 21.31 μg/mL. For each treatment time group, the extracellular polysaccharide content was lowest after the US − SAEW treatment. After 60 s of US − SAEW treatment, the content was only 1.17 μg/mL. The reduction of extracellular polysaccharide proved that the Lm biofilms was removed, thus indicating that the US − SAEW treatment had the best biofilm removal effect in each group, the decrease trend of extracellular exopolysaccharides was also consistent with that of the total number of colonies (Fig. 5). Similar results were reported that phenyl lactic acid can destroy the biofilm structure of Lm by acting on extracellular polysaccharide and extracellular protein [38].

**Fig. 6:**
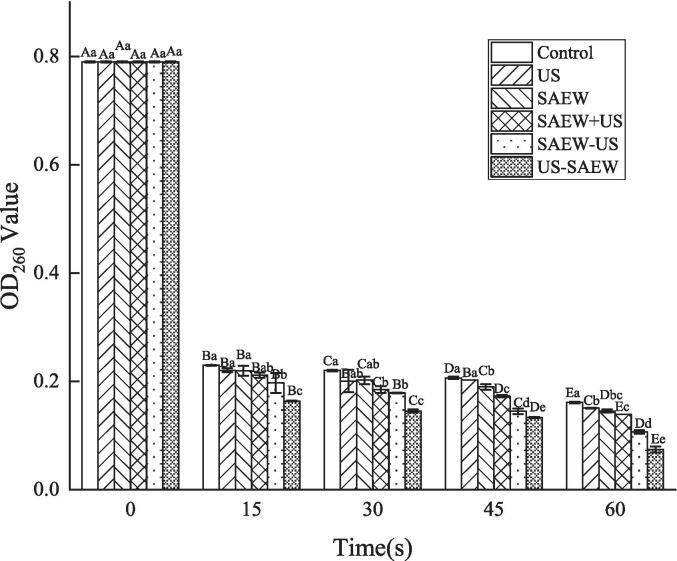
The changes in extracellular DNA of *Listeria monocytogenes* biofilms after different treatments. Capital letters represent significant differences between different processing times. Lower case letters represent significant differences between different treatments (P < 0.05). US, ultrasound. SAEW, slightly acidic electolyzed water.

DNA is the basis for the normal physiological activities of bacteria. If their DNA is damaged, bacteria cannot grow and reproduce normally, meaning that a rapid decline in DNA content destroys the self-assembly of the biofilm structure community and the transfer of genes between cells [37], [39], [40]. Nucleic acid is a substance that absorbs light with a wavelength of 260 nm, and the optical density value of the supernatant at 260 nm represents the absorbance value of the nucleic acid. For the 0 s treatment groups, the OD_260_ value was 0.790. With increasing treatment time, the OD_260_ value gradually decreased, a trend corresponding to the bactericidal effect of each treatment. In each treatment time group, the amount of DNA present was the lowest after the US − SAEW treatment. In the 60 s treatment group, the OD_260_ value from the US − SAEW treatment was only 0.074, confirming that US − SAEW had the best biofilm removal effect in each group (Fig. 6). Extracellular DNA are key components of EPS. It is released extracellularly through active bacterial secretion and cytolytic action, adsorbs to the bacterial cell surface, and extends outwards, thus promoting biofilm formation. In addition, it can provide nutrients for bacterial metabolism, act as a “gene store” for gene-level transmission, cross-link with extracellular proteins and polysaccharides, and maintain the biofilm spatial structure [41]. When treated with US − SAEW, the bacteria died or the extracellular polymer structure was destroyed, resulting in a decrease in the content of extracellular DNA, and the bacteria without extracellular DNA lost drug resistance, increasing the impact of US − SAEW treatment on the bacteria itself. The study of Banerji et al. also obtained similar results that spices inhibit biofilm formation in Lm by reducing the release of extracellular DNA [42]. The change trend of this value is consistent with the change trend of the previous results, which preliminarily suggests that the most important reason for the effectiveness of US − SAEW treatment in removing biofilm is the destruction of EPS of biofilm.

In summary, the US − SAEW treatment showed optimal Lm biofilm removal of all treatment groups in this study. The formation and collapse of cavitation bubbles weakens cell walls and breaks chemical bonds in cell membranes, which could have contributed to the ability of SAEW to penetrate the Lm cells. Due to the strong oxidizing ability of SAEW, the permeability of cell membranes as well as intracellular nucleic acids and proteins, causing Lm death. Also, US − SAEW treatment destroys the extracellular polymer of the biofilm, which is one of the reasons for the removal of the biofilm. In this study, the results showed that US − SAEW treatment had the best effect on reducing the number of biofilm cells. In previous studies, we found that the combination of acid-alkali electrolyzed water treatment for 10 min resulted in a 99.92 % sterilization rate. Moreover, the residual amount of extracellular nucleic acid was measured using luciferase labelling, and it was observed that the extracellular DNA was reduced during treatment [29]. However, in the present study, 100 % sterilization rate was reached by US-SAEW method for 60 s and the extracellular DNA was also reduced. It suggests that the method greatly reduces the treatment time and saves cost.

Food processing equipment that is difficult to clean and sanitize, such as conveyor belts and rubber seals, and facility infrastructure that is less frequently cleaned and sanitized, such as floors and drains, are some of the most common sites for biofilm buildup and are thus potential sources of Lm contamination in food [43]. In the food production process, glass is usually used to make a variety of food containers, such as sauce bottles, wine bottles, canning jar. Because the transparent characteristics facilitate consumers to observe the state of food. These potential factors can lead to contamination of glass containers or tableware, which increases the risk of food product contamination. Consumption of contaminated food can cause harm to the body, leading to serious food safety issues. Consequently, it is important to sterilize glassware using US − SAEW treatment. Lm can form biofilms on different surfaces, including stainless steel, glass, polystyrene, polyethylene and rubber [44], [45]. Therefore, the next step in our research program is to build on previous studies by culturing Lm on materials commonly used in industrial equipment, such as stainless steel, wood, and polypropylene. This will be followed by quantifying how much Lm biofilm can be removed from those materials, determining the extracellular polymeric substance content, and observing morphological changes such as the degree of damage to the Lm biofilm after treatment. At present, the standards and technical parameters for the application of US sterilization treatment have not been clearly defined and standardized. Sterilization using US treatment alone is limited, resulting in the existence of sublethal cells that increase safety risks [46], [47]. As a broad-spectrum and efficient fungicide, SAEW has been applied to the sterilization and disinfection of various foods and utensils. However, the bactericidal effectiveness of SAEW is correlated with a number of factors (including available chlorine concentration, treatment time, product type, etc.). In the process of US − SAEW treatment, the specific conditions under which the frequency and time of US use, the available chlorine concentration of SAEW and the physicochemical indexes affect the clearance effect of Lm biofilm need to be further studied [48]. Excessive available chlorine concentration in SAEW and inappropriate US treatment conditions will cause corrosion to food processing equipment to a certain extent, and it will also adversely affect the quality of food itself when it is used to treat food raw materials. Therefore, determining an effective available chlorine dose and appropriate sonication conditions for US − SAEW treatment is currently a challenge in the biofilm removal studies.

To better remove the biofilm from the glass food containers, the Lm biofilm could be eluted using the treatment outlined in this study, as well as sterilizing and removing biofilm. The effectiveness of this method in removing bacterial biofilms from other different material surfaces of food processing equipment will continue to be investigated. The effective dosage of available chlorine and suitable ultrasound conditions for US − SAEW treatment will be determined. Ultimately, it is hoped to provide a more refined method for the more efficient and environmentally friendly removal of biofilms, which is essential for the development of the food industry.

## 4 Conclusions

In this study, the efficacy of the control, US, SAEW, SAEW + US, SAEW − US and US − SAEW treatments for removing Lm biofilms was compared using the plate counting method. Crystal violet staining was used to observe the effect of the six treatment methods on the amount of Lm biofilm, and to allow a preliminary evaluation of the effectiveness of the treatments in removing Lm biofilms. The number, appearance, and ultrastructure of Lm biofilms treated using the six treatment methods were also examined using scanning electron microscopy to determine their effect on biofilm morphology. Finally, the bactericidal effect of the six treatments on the Lm biofilms was determined by comparing the changes they caused in the EPS. The results showed that, for all treatment durations, the US − SAEW treatments led to the lowest number of residual colonies. The crystal violet staining and scanning electron microscopy also showed that the lowest number of residual bacteria remained after US − SAEW treatment, with the trend of changes in extracellular polymers being consistent with the bactericidal results. This showed that the US − SAEW method was best at removing Lm biofilms. Consequently, the US − SAEW treatment is a more efficient and environmentally friendly method for the removal of biofilms.

## CRediT authorship contribution statement

**Hongrui Ren:** Writing – original draft, Methodology, Investigation. **Yu Quan:** Writing – original draft, Methodology, Investigation. **Shaokang Liu:** Investigation, Formal analysis. **Jianxiong Hao:** Writing – review & editing, Resources, Investigation, Funding acquisition.

## Funding

This work was supported by 10.13039/501100001809National Natural Science Foundation of China [grant number 31972170]; and 10.13039/501100017697Hebei Province Outstanding Youth Fund [grant number C2018208085].

## Declaration of competing interest

The authors declare that they have no known competing financial interests or personal relationships that could have appeared to influence the work reported in this paper.
